# Chemical biology-whole genome engineering datasets predict new antibacterial combinations

**DOI:** 10.1099/mgen.0.000718

**Published:** 2021-12-07

**Authors:** Arthur K. Turner, Muhammad Yasir, Sarah Bastkowski, Andrea Telatin, Andrew Page, Mark Webber, Ian Charles

**Affiliations:** ^1^​ Quadram Institute Bioscience, Norwich Research Park, Norwich NR4 7UQ, UK; ^2^​ University of East Anglia, Norwich Research Park, Norwich, NR4 7TJ, UK

**Keywords:** chemical, genomics, TraDIS, TIS, antibiotics, combinations

## Abstract

Trimethoprim and sulfamethoxazole are used commonly together as cotrimoxazole for the treatment of urinary tract and other infections. The evolution of resistance to these and other antibacterials threatens therapeutic options for clinicians. We generated and analysed a chemical-biology-whole-genome data set to predict new targets for antibacterial combinations with trimethoprim and sulfamethoxazole. For this we used a large transposon mutant library in *Escherichia coli* BW25113 where an outward-transcribing inducible promoter was engineered into one end of the transposon. This approach allows regulated expression of adjacent genes in addition to gene inactivation at transposon insertion sites, a methodology that has been called TraDIS-*Xpress*. These chemical genomic data sets identified mechanisms for both reduced and increased susceptibility to trimethoprim and sulfamethoxazole. The data identified that over-expression of FolA reduced trimethoprim susceptibility, a known mechanism for reduced susceptibility. In addition, transposon insertions into the genes *tdk*, *deoR*, *ybbC*, *hha*, *ldcA*, *wbbK* and *waaS* increased susceptibility to trimethoprim and likewise for *rsmH*, *fadR*, *ddlB*, *nlpI* and *prc* with sulfamethoxazole, while insertions in *ispD*, *uspC*, *minC*, *minD*, *yebK*, *truD* and *umpG* increased susceptibility to both these antibiotics. Two of these genes’ products, Tdk and IspD, are inhibited by AZT and fosmidomycin respectively, antibiotics that are known to synergise with trimethoprim. Thus, the data identified two known targets and several new target candidates for the development of co-drugs that synergise with trimethoprim, sulfamethoxazole or cotrimoxazole. We demonstrate that the TraDIS-*Xpress* technology can be used to generate information-rich chemical-genomic data sets that can be used for antibacterial development.

## Data Summary

Nucleotide sequence reads data fastq files are available from: http://www.ebi.ac.uk/arrayexpress/experiments/E-MTAB-9305.

Other supplementary data. The complete data set for trimethoprim (TMP) and sulfamethoxazole (SUL) for *q*-values<0.01 is included in Table S1 (available in the online version of this article).

This provides the log_2_fold-change (log_2_FC), log_2_counts-per-million (log_2_CPM) and *q*-values for each gene, or 5prime and 3prime gene regions (each provided on separate sheets). For the 5prime and 3prime gene regions, the number of sequence reads data is split into the forward (for) and reverse (rev) DNA strands to allow transposon orientation to be considered. For genes, forward and reverse data are combined (comb). Where the locus is given as numbers (e.g. ‘1924179_1924263’), these are coordinates that indicate a possible locus of interest for which there are no immediately adjacent annotated features.

‘#N/A,’ not applicable, indicates that insufficient nucleotide sequence reads were located to the feature for the AlbaTraDIS analysis software to calculate parameters.

Table headings include the locus, and for the 5prime and 3prime gene data, the coding strand (Co_Str); ‘2_’, ‘1_’, ‘0.5_’ and ‘0.25_’ indicate that the data are from 2, 1, 0.5 and 0.25× MIC growth conditions respectively. Thus, for example, ‘0.5_log_2_FCfor’ is the log_2_fold-change data at 0.5× MIC for the forward strand, and 2_qrev is the *q*-value at 2× MIC for the reverse strand.

**Impact Statement**

The spread of antibiotic resistance has led to a need for new treatments for infections. One option is to identify novel and more potent combinations of existing drugs already approved for other uses. However, many different drugs require a very large number of test combinations. By using a genomics technology, we have assayed every gene of the bacterium *Escherichia coli* for a role in susceptibility to the antibiotics trimethoprim and sulfamethoxazole, simultaneously for the first time. This provides an amount of information on the effects of these antibiotics on the bacterial cell not previously achieved. A relatively small number of genes were found that contribute to susceptibility to these antibiotics, whilst it was also confirmed that the majority of genes had no bearing on susceptibility. Of the genes that contribute to susceptibility, some have been found previously to be targeted by compounds that provide more potent antibiotic combinations with trimethoprim, confirming that this genomics technology can help predict new antibiotic combinations. This technology is applicable to the identification of new antibiotic targets and novel combinations for any antibiotic. Thus, it is demonstrated that this novel genomics technology will provide a significant step change when applied to antibiotic development.

## Introduction

The pyrimidine antibiotic trimethoprim is used widely to treat urinary and respiratory tract infections. It is one of the most prescribed antibiotics, accounting for about 11 % of all antibiotic prescriptions [1]. In *E. coli* and other susceptible bacteria, trimethoprim is an inhibitor of the *folA* gene product, dihydrofolate reductase (DHFR), inhibiting the conversion of dihydrofolate to tetrahydrofolate, a methyl donor required for the biosynthesis of thymidylate, pyrimidines, purines and methionine. Trimethoprim poisoning of bacteria thus inhibits synthesis of DNA [2]. In addition to incorporation into proteins, methionine is also required for the biosynthesis of S-adenosyl methionine, a methyl donor required for the biosynthesis of methylated derivatives, including methyltransferases, tRNA and protein modifications, and amino acid biosynthesis.

One step in the biosynthesis of tetrahydrofolate includes the formation of dihydropteroate (DHP) from pteridine diphosphate and *para*-aminobenzoic acid (*p*ABA), in a reaction catalysed in *E. coli* by the *folP* gene product, dihydropteroate synthase (DHPS). The antibiotic sulfamethoxazole is a *p*ABA analogue that acts by being incorporated into this reaction instead of *p*ABA, converting pteridine diphosphate into the dead-end, non-metabolite, dihydropterin-sulfamethoxazole. This starves the cell of dihydropteroate and, consequently, tetrahydrofolate [3]. Sulfamethoxazole is rarely used alone as an antibiotic due to bacterial resistance and availability of more active and less toxic alternatives. But due to it inhibiting folate synthesis, it is used in combination with trimethoprim as co-trimoxazole, to treat urinary and respiratory tract infections, otitis media, nocardiosis, pneumonia caused by *Pneumocystis jirovecii*, and toxoplasmosis.

*E. coli* is the major cause of urinary tract infections, and, as with many antibiotics, resistance to trimethoprim and cotrimoxazole has increased [4–12]. In most cases this is due to concurrent acquisition by genetic transfer of alternative DHFR and DHPS enzymes that have reduced binding affinity for trimethoprim and sulfamethoxazole, encoded by *dfrA* and *sul* alleles respectively. Consequently, by the year 2000, due to increasing resistance, cotrimoxazole was used less frequently for the treatment of urinary tract infections [13]. As well as trimethoprim-insensitive *dfrA* genes, point mutations within, and over-expression of, *folA* have been identified as resistance mechanisms in the laboratory and in occasional clinical isolates [3, 14–18].

**Fig. 1.**
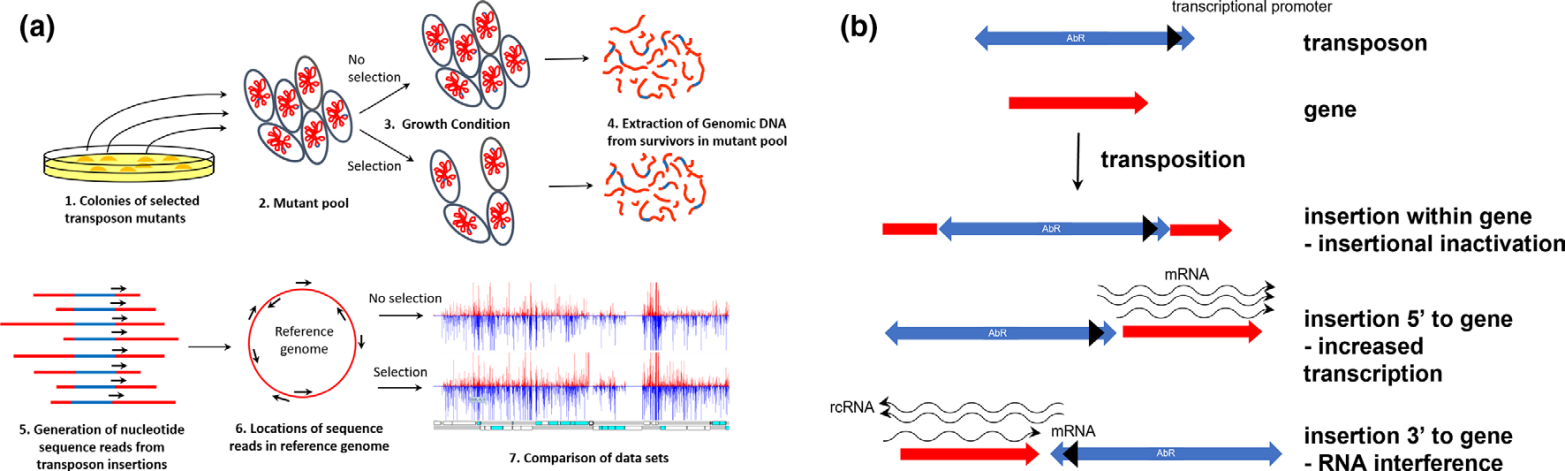
(a) Steps in the generation of data using TraDIS-*Xpress*. Following introduction of the transposon into the bacteria, transposon mutant colonies are grown on nutrient agar medium with selection for the transposon-encoded resistance determinant (1). Colonies are harvested from agar surfaces by suspending in nutrient broth and scraping the mutants into a large pool (2). The mutant pool is then subjected to a growth condition of interest and an appropriate control condition (3), then genomic DNA is extracted (4). Using a customised nucleotide sequencing protocol, sequence reads are then generated from the known sequences within the end of the transposon into the nucleotides surrounding the transposon insertion site (5). Where the sequence reads match the sequence of a reference genome sequence locates the transposon insertion sites (6), and the number of reads at any site reflects the number of mutants. Comparison of data sets for different growth conditions indicates those mutations which confer a competitive change, thereby identifying genes relevant to the growth condition of interest (7). (b) Likely consequences following insertion into the bacterial genome of a transposon incorporating an outward transcribing promoter. The transposon (blue) used for TraDIS-*Xpress* encodes an antibacterial resistance determinant (AbR) to enable selection of transposon mutants and incorporates an outward transcribing promoter (black arrowhead). If the transposon inserts within a gene (red) the result is likely to be insertional inactivation of the gene. Insertion 5’ to a gene can result in altered transcription, and insertion 3’ to a gene can result in altered expression due to RNA interference (RNAi) of reverse complimentary RNA (rcRNA) with the native mRNA.

Previously, we have developed TraDIS-*Xpress* as a means of assaying every gene in the *E. coli* genome for a role under chosen growth conditions [19]. This genomics technology uses very large transposon mutant libraries and modifications to ultra-high throughput nucleotide sequencing to generate sequence reads from within the end of the transposon into the adjacent insertion sites (Fig. 1a). Matching the nucleotide sequence of the reads with that of a reference genome allows the simultaneous identification of insertions sites for hundreds of thousands of different mutants simultaneously, thus providing a quantitative measure of mutant fitness following growth in a chosen condition (Fig. 1a). By including an inducible outward transcribing promoter in the transposon end, as well as assaying genes by insertional inactivation, genes are also assayed by altered transcription (Fig. 1b). This may operate by altering gene expression through transcriptional changes, or by RNA interference mechanisms (Fig. 1b).

Thus, roles for essential genes, into which transposon insertions are lethal and are therefore absent from the mutant collection, may be inferred from changes in abundance of adjacent transposon insertions. By generating millions of nucleotide sequence reads specifically from the transposon ends, the reads provide a surrogate measure of the relative abundance of the different mutants present. In addition, by using several hundred thousand different mutants there is, on average, a representative mutant every few nucleotides along the genome, and so the whole genome is assayed at a very high-level of resolution.

For this work we have applied TraDIS-*Xpress* using a very large *E. coli* transposon mutant collection to identify candidate genes that contribute to reduced or increased susceptibility to trimethoprim or sulfamethoxazole. This strategy aimed to provide an indication of novel mechanisms that may contribute to resistance and susceptibility for *E. coli* and other susceptible bacteria, which in turn can indicate how to prevent resistance from arising and how to make bacteria more susceptible to these and possibly other antibiotics. In addition, such genomic scale information can indicate the broader effects that antibiotics have on bacteria (e.g. identify modes of entry and efflux) and provides clues to improve our understanding of bacterial cell biology.

## Methods

### TraDIS-*Xpress* transposon mutant library

The high density TraDIS-*Xpress* transposon mutant library has been described previously [19]. It was constructed using *E. coli* strain BW25113, the same strain as that used for construction of the single gene knockout mutant, ‘Keio,’ collection [20], thus allowing for easier verification of TraDIS-*Xpress* results. The transposon used to construct the mutant collection is a mini-Tn*5*-derivative with the *aph*(3’)-Ia gene coding for kanamycin resistance and incorporating an outward-transcribing IPTG-inducible *tac* promoter, thus providing mutation by insertional inactivation and by altered expression of adjacent genes. All the transposon mutants are collected and stored as a single pool.

### Growth conditions with trimethoprim and sulfamethoazole

Approximately 10^7^ c.f.u. of transposon mutant library was grown in LB broth supplemented with trimethoprim at 2, 1, 0.5 or 0.25 mg l^−1^, or sulfamethoxazole at 4, 2, 1 or 0.5 g l^−1^, and without any antibiotic. These concentrations are approximately equivalent to 2, 1, 0.5 and 0.25× the MIC respectively of each drug. For each antibiotic concentration the transposon outward-transcribing promoter was either uninduced or induced with IPTG at 0.2 mM, or 1 mM. For data input into the ‘AlbaTraDIS’ software ([21]; see below), each condition was performed in duplicate.

### Generation of nucleotide sequence reads using TraDIS-*Xpress*

Following growth under the experimental conditions, genomic DNA was extracted from bacteria using the *Quick*-DNA Fungal/Bacterial 96 kit (Zymo Research), fragmented, and nucleotide sequence reads generated specifically from the transposon promoter ends using a Nextera 550 sequencing machine (Illumina) as described previously [19].

### TraDIS-*Xpress* nucleotide sequence analysis

Nucleotide sequence data were analysed using the BioTraDIS (version 1.4.1) [22] and AlbaTraDIS software (version 0.0.5) developed for TraDIS-*Xpress* analysis and recently described [21]. Briefly, BioTraDIS was used to locate sequence reads in the BW25113 reference genome (CP009273) using SMALT, thereby identifying the site of transposon insertion. For all growth conditions, between three million and 30 million sequence reads were located in the reference genome. In order to simplify data analysis and presentation, data for different IPTG concentrations used for induction of the outward transcribing promoter were amalgamated. The number of nucleotide sequence reads that locate to a single point is a semi-quantitative measure of the number of mutants with insertions at that point. The resulting insertion plots can be visualised for manual inspection using the ‘Artemis’ software [23]. Then for each gene, and for 198 bp 5-prime and 3-prime of each gene, AlbaTraDIS compared data from condition and no drug control and calculated the number of transposon inserts in the ‘forward,’ ‘reverse’ and both (‘combined’) orientations. This information is used to identify genes and their adjacent regions that had significantly changed insertion patterns between condition and control. Information from gene adjacent regions can indicate the effect of insertion of the transposon with its outward-transcribing promoter.

Output from the AlbaTraDIS software includes: a list of genes with significantly changed insertion pattern for a condition compared to the controls (gene_report), as well as the fold change in the number of nucleotide sequence reads between control and condition for each gene, converted to log_2_ (log_2_FC); the number of reads that located to the gene (counts) per million of total reads obtained for the sample, converted to log_2_ (log_2_CPM); a *p*-value of statistical significance; and a *q*-value (the *p*-value adjusted for the false discovery rate). The data for genes presented in Table 1 had a *q*-value <0.0001 and log_2_CPM>6. The network analysis includes genes only which had *q*<0.001 and log_2_CPM>5 (Fig. 1).

### Network analysis

In order to create the network (Fig. 1), the ‘gene_report’ output of AlbaTraDIS (albatradis script) was summarised for all concentrations of trimethoprim and sulfamethoxazole using the albatradis-presence_absence script. The network represents all genes that showed a significant change in mutants for the different conditions. R (version 3.6.3) [22] was used to customise the network by annotating edges with log_2_FC and Gephi [24] and Cytoscape [25] were used to draw the network. In order to simplify the network and include only the most significant data, genes were included for which *q*<0.001 and log_2_CPM>5.

### Relative MIC determinations

These were performed using 96-well microplates with 200 µl LB broth per well. As well as two-fold serial dilution, 0.8-fold serial dilution of trimethoprim (0.8, 0.64, 0.512, 0.41, 0.328, 0.262, 0.21 mg l^−1^) were used. Approximately 10^4^ c.f.u. of bacteria were added and incubated at 37 °C overnight. The concentration of antibiotic at which the growth was inhibited >90% based on optical density was taken as the MIC. Antibacterial synergy between trimethoprim and 3′-azido-3′-deoxythymidine (AZT) was assessed similarly employing combinations of concentrations of both.

### Competition assays

In order to determine the relative fitness between mutants chosen from the Keio mutant collection and the parent strain *E. coli* BW25113, competition assays were performed in LB broth without trimethoprim or supplemented with 0.25 mg l^−1^ and inoculated with ~6×10^3^ c.f.u. ml^−1^ of BW25113 and a similar number of the Keio mutant to be tested. The Keio collection includes duplicates of each mutant, so these assays were performed once and separately for each pair of mutants. Immediately after inoculation, 5 µl of undiluted and 10^−1^ dilution of the cultures were spotted onto LB agar and in replicate onto LB agar supplemented with kanamycin at 30 mg l^−1^ to confirm the proportion of kanamycin resistant mutants. This was repeated after 6 h incubation at 37 °C using ten-fold serial dilutions to 10^−5^, and after 20 h incubation using dilutions to 10^−7^. The proportion of mutants and parent strain was then determined at the different trimethoprim concentrations from the c.f.u.

## Results

The transposon mutant library has been described previously [19], exists as a single pool and is estimated to consist of at least 380 thousand mutants. Approximately 10^7^ c.f.u. of this mutant pool were grown in each different concentration of trimethoprim or sulfamethoxazole, or without these antibiotics to provide control data. In addition, each antibiotic condition was performed with two different concentrations of IPTG or without IPTG to provide different levels of induction of the outward-transcribing promoter that was incorporated into the end of the transposon. However, to simplify the data analysis and presentation, data obtained for the different induction levels have been amalgamated. Also, in clinical use, trimethoprim and sulfamethoxazole are most commonly used in a combined formulation (‘Cotrimoxazole’), but they were analysed separately in the experiments described here so that any susceptibility mechanisms that were identified could be ascribed specifically to one or other of the antibiotics.

**Fig. 2.**
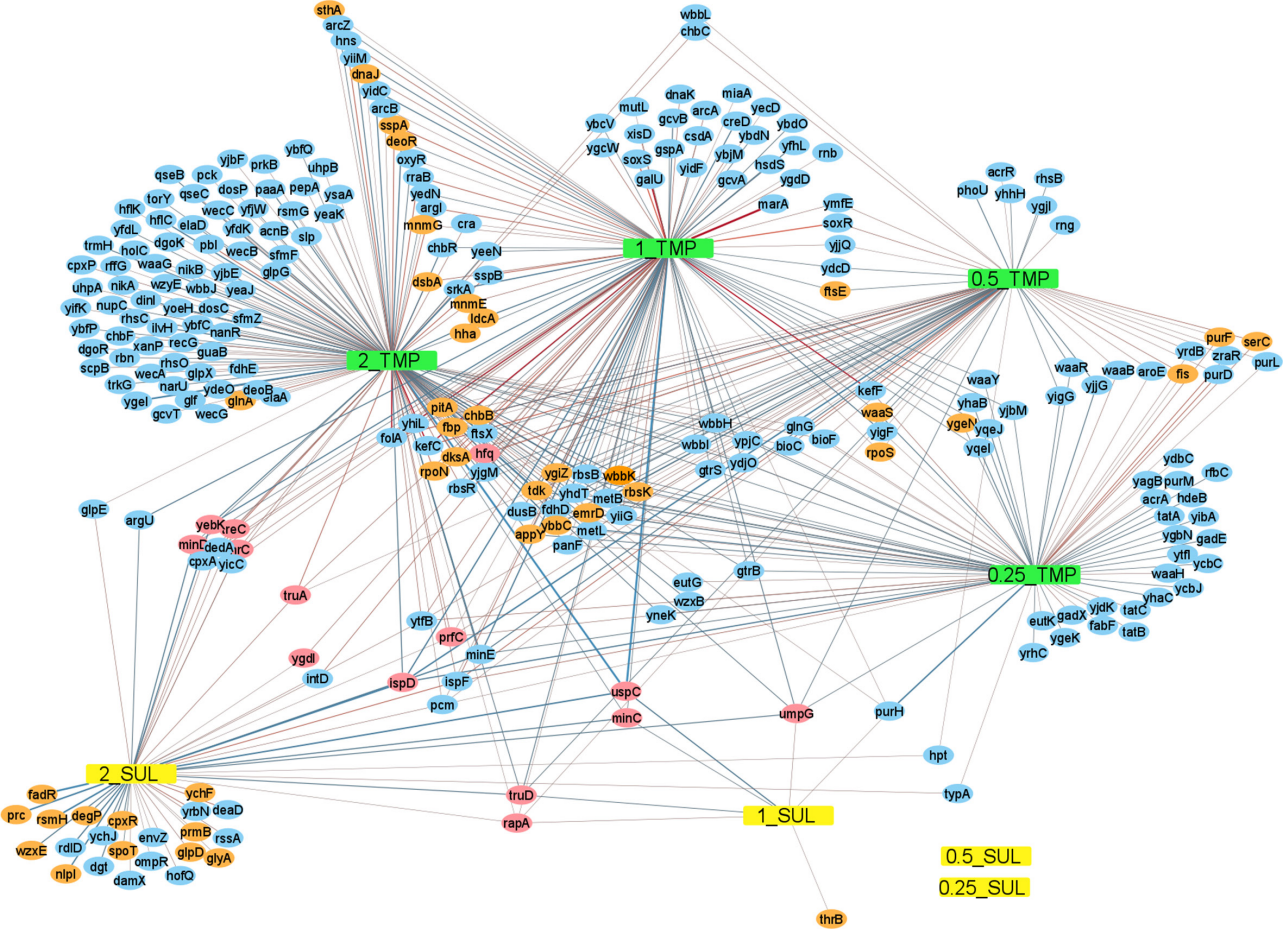
Network analysis of genes involved in susceptibility to trimethoprim and sulfamethoxazole based on insertional inactivation. Genes for which *q*<0.001 and log_2_CPM>5 are included. TMP, Trimethoprim, SUL, sulfamethoxazole. 2_, 1_, 0.5_ and 0.25_ indicates 2, 1, 0.5 and 0.25× MIC respectively. Genes on a green or grey background gave the most statistically significant result (*q*<0.0001, log_2_CPM>6) and are also listed in Table 1, grey being involved in both trimethoprim and sulfamethoxazole susceptibility and green being involved in susceptibility to only one of these. Genes on a blue background were less statistically significant (*q*<0.001, log_2_CPM>5) and are not listed in Table 1. Red connectors indicate an increase in mutants, and blue a decrease, with the width proportional to the fold change.

Filtering the data for differences in mutant insert patterns between the antibiotic test and control conditions based on statistical significance and sufficient numbers of reads (*q*<0.01 and log_2_CPM>4) generated a list of 416 candidate genes of relevance to growth in the presence of trimethoprim or sulfamethoxazole. Using the same statistical filter, no genes were identified that were of relevance to growth in 0.25× or 0.5× MIC sulfamethoxazole, indicating that these concentrations lead to no significant difference in the numbers of mutants compared to growth without sulfamethoxazole, and also that these data filtering parameters provide a robust cut-off for identifying statistically significant changes. A network analysis generated from the more statistically significant genes only (those genes for which *q*<0.001 and log_2_CPM>5) illustrates how some genes are relevant to only a single condition, whilst others are relevant to several, including 14 relevant to both trimethoprim and sulfamethoxazole (Fig. 2). Table 1 lists the 58 genes for which the data showed most significance (*q*<0.0001, log_2_CPM>6), where a positive log_2_FC indicates mutants that increased in numbers over the course of the experiment and a negative log_2_FC a decrease in mutant numbers. These values therefore provide a measure of relative mutant fitness.

**Table 1.** Genes within which transposon insertions result in gain or loss of susceptibility to trimethoprim or sulfamethoxazole

Category	Gene*	TMP log_2_FC†	SUL log_2_FC	Functions
Regulators	*dksA*	1.8/2.8/2.2/0	0/0	transcriptional regulator of rRNA transcription, dnaK suppressor protein
	*fis*	0/0/2.6/1.8	0/0	global DNA-binding transcriptional dual regulator
	*sspA*	1.5/1.7/0/0	0/0	stringent starvation protein A, RNAP-associated acid-resistance protein
	*appY*	−1.3/−1.2/−1/−1.3	0/0	global transcriptional activator; DLP12 prophage
	*cpxR*	0/0/0/0	−1.4/0	response regulator in two-component regulatory system with CpxA
	***yebK***	−1.2 / −1.2/0/0	−1.4/0	putative DNA-binding transcriptional regulator
	*spoT*	0/0/0/0	−1.6/0	(p)ppGpp synthetase II/ guanosine-3',5'-bis pyrophosphate 3'-pyrophosphohydrolase
	*hha*	−1.7/−1.3/0/0	0/0	modulator of gene expression, with H-NS
	***uspC***	−1.8/−1.4/0/0	−1.4/−1.2	universal stress protein
	*nlpI*	0/0/0/0	−2.1/0	lipoprotein involved in osmotic sensitivity and filamentation
	*deoR*	−2.4/−1.7/0/0	0/0	deoxyribose operon transcriptional repressor; repressor of nupG and tsx
	*fadR*	0/0/0/0	−3.1/0	fatty acid metabolism regulon transcriptional regulator
RNA	*rpoN*	2.4/2.6/2.5/0	0/0	RNA polymerase, sigma 54 (sigma N) factor
polymerase	***rapA***	1.7/1.7/1/0	0.8/1.1	RNA polymerase recycling factor ATPase; ATP-dependent RNA translocase
	*rpoS*	0/1.4/1.5/1	0/0	RNA polymerase, sigma S (sigma 38) factor
RNA	***truA***	1.7/1.6/1.2/0	1.7/0	tRNA pseudouridine(38-40) synthase
modification	*mnmE*	1.7/1.5/0/0	0/0	tRNA U34 5-methylaminomethyl-2-thiouridine modification GTPase
	*mnmG*	1.3/1.2/0/0	0/0	5-methylaminomethyl-2-thiouridine modification at tRNA U34
	***truD***	−1/−1.2/−0.9/0	−1.4/0	tRNA(Glu) pseudouridine(13) synthase
	*rsmH*	0/0/0/0	−3.3/0	16S rRNA m(4)C1402 methyltransferase, sAM-dependent
Nucleoside/	***dcd***	4.7/4.3/4.3/3.8	4.3/0	2'-deoxycytidine 5'-triphosphate deaminase
nucleotide	*sthA*	1.8/1.5/0/0	0/0	pyridine nucleotide transhydrogenase, soluble
metabolism	*purF*	0/1/1.3/1.3	0/0	amidophosphoribosyltransferase
	***umpG***	−1 / −1/−0.8/−0.8	−1.3/0	broad specificity 5'(3')-nucleotidase and polyphosphatase
	*tdk*	−3.9/−2.8/−2/−1.4	0/0	thymidine kinase/deoxyuridine kinase
Amino acid	*glnA*	2.5/2.3/2.2/0	0/0	glutamine synthetase
biosynthesis	*glyA*	0/0/0/0	1.5/0	serine hydroxymethyltransferase
	*serC*	0/0/1.5/1.4	0/0	3-phosphoserine/phosphohydroxythreonine aminotransferase
	***thrC***	1.3/1.2/0/0	1.3/0	l-threonine synthase
	*thrB*	0/0/0/0	1/1.2	homoserine kinase
Others	*fbp*	2.1/1.7/1.3/0	0/0	fructose-1,6-bisphosphatase I
	*prmB*	0/0/0/0	2/0	N5-glutamine methyltransferase
	***prfC***	2/1.8/1.2/1.3	1.5/0	peptide chain release factor RF-3
	***hfq***	1.9/1.9/1.7/0	1.8/0	global sRNA chaperone; HF-I, host factor for RNA phage Q beta replication
	*dnaJ*	1.9/1.9/1/0	0/0	chaperone Hsp40, DnaK co-chaperone
	*dsbA*	1.9/1.7/0/0	0/0	periplasmic protein disulfide isomerase I
	***ygdI***	1.6/1.8/0/0	1.7/0	DUF903 family verified lipoprotein
	*rbsK*	1.5/1.3/1.2/1	0/0	ribokinase
	*pitA*	1/1.3/1.5/0	0/0	phosphate transporter, low affinity; tellurite importer
	***treC***	1.5/1.2/0/0	1.4/0	trehalose-6-P hydrolase
	*ychF*	0/0/0/0	1.5/0	catalase inhibitor protein; ATPase, K+-dependent, ribosome-associated
	*emrD*	1.3/1.2/1.2/1.2	0/0	multidrug efflux system protein
	*glpD*	0/0/0/0	1.2/0	sn-glycerol-3-phosphate dehydrogenase, aerobic, FAD/NAD(P)-binding
	*ftsE*	0/−1.2/−1.4 / −1.2	0/0	putative ABC superfamily transporter ATP-binding subunit
	*ygiZ*	−1.2/-1.4/−1.3 / −1.4	0/0	inner membrane protein
	*degP*	0/0/0/0	−1.4/0	serine endoprotease (protease Do), membrane-associated
	*ygeN*	0/−1.4/0/−1.5	0/0	pseudogene
	*chbB*	−1.5/−1.2/−1.3/0	0/0	N,N'-diacetylchitobiose-specific enzyme IIB component of PTS
	*waaS*	0 / −1/-1.2/-1.5	0/0	lipopolysaccharide core biosynthesis protein
	***minC***	−1.5/−1.6/0/0	−1.5/−1.2	cell division inhibitor
	***minD***	−1.4/−1.3/0/0	−1.6/0	membrane ATPase of the MinC-MinD-MinE system
	*wbbK*	−1.6/−1.2/−1.1 / −1.2	0/0	lipopolysaccharide biosynthesis protein
	*prc*	0/0/0/0	−2/0	carboxy-terminal protease for penicillin-binding protein 3
	*wzxE*	0/0/0/0	−2.1/0	O-antigen translocase
	*ybbC*	−2.1/−1.8/−1.4 / −1.7	0/0	putative immunity protein
	*ldcA*	−2.1/−2.2/0/0	0/0	murein tetrapeptide carboxypeptidase; ld-carboxypeptidase A
	***ispD***	0/−2.2/−2.6 / −2.1	−2.7/0	4-diphosphocytidyl-2C-methyl-d-erythritol synthase
	*ddlB*	0/0/0/0	−2.7/0	d-alanine:D-alanine ligase

*Genes in bold type were relevant to growth with trimethoprim and with sulfamethoxazole.

†TMP, trimethoprim; SUL, sulfamethoxazole. Log_2_FC values are shown for 2, 1, 0.5 and 0.25× MIC trimethoprim, and 2× MIC and 1× MIC for sulfamethoxazole (for which 0.5 and 0.25× MIC conditions failed to yield any statistically significant data) with / as a separator. Any condition that yielded no statistically significant data for a gene in the table are shown by zeros.

Insertion mutations within approximately half of these 58 genes conferred a growth advantage, whilst for the other half a growth disadvantage was conferred in the presence of trimethoprim or sulfamethoxazole (Table 1). For 15 of these genes, insertion mutations conferred a phenotype following growth in both trimethoprim and in sulfamethoxazole.

**Fig. 3.**
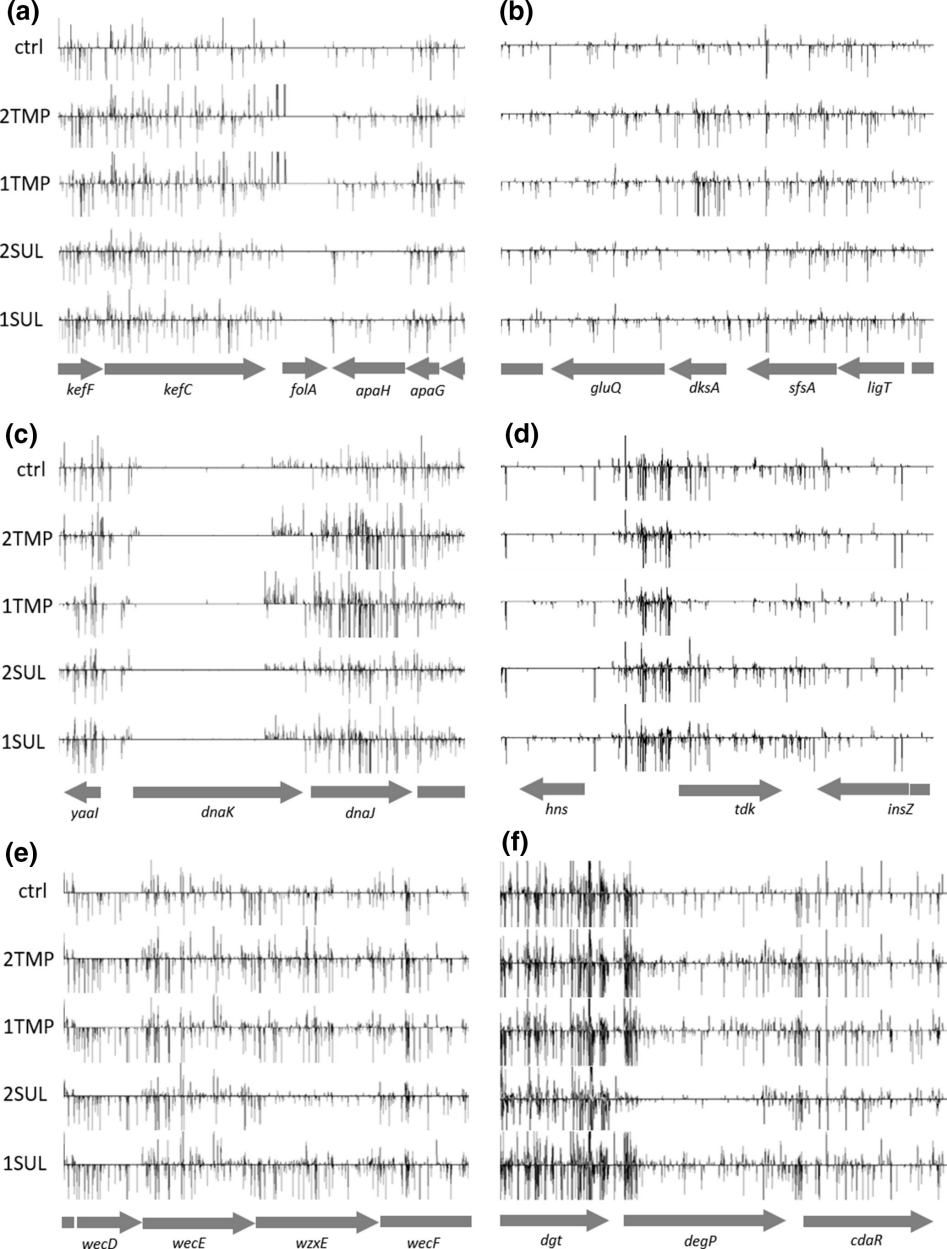
Visualisation of TraDIS-*Xpress* data for *folA*, *dksA*, *dnaKJ*, *tdk*, *wzxE*, *degP* and their adjacent genes. Along the bottom of each panel the relative location of each gene is indicated. Above this are five horizontal frames, one control (ctrl) and 2 x MIC and 1× MIC each for trimethoprim and sulfamethoxazole (2TMP, 1TMP, 2SUL and 1SUL respectively). Vertical lines within these frames indicate transposon insertion sites, and the height of these reflects the number of reads that located to each site which is an indicator of the number of insertion mutants. Vertical lines above the axis indicate reads that located to the forward strand such that the outward-transcribing promoter in the transposon end transcribes in the left to right direction, and those below the axis indicate transposon insertions in the opposite orientation. Only one of the duplicate data sets for each condition is shown. (a) *folA* shows transposon insertions upstream and transcribing into *folA* following growth with trimethoprim but no significant changes across this entire region with sulfamethoxazole compared to growth without antibiotic. Note that there are no sequence reads locating within *folA*, indicating that this is an essential gene and would not have been assayed without the transposon outward-transcribing promoter. (b) *dksA* shows an increase in mutants following growth in 2TMP and 1TMP only. (c) *dnaKJ* shows increased mutants following growth in 2TMP and 1TMP only, with insertions into the 3’-quarter of *dnaK* only and predominantly in one orientation. (d) *tdk* shows fewer mutants following growth in 2TMP and 1TMP only. (e) and (f) *wzxE* and *degP* show fewer mutants following growth in 2SUL.

### Growth of knock-out mutants in competition experiments confirms the TraDIS-Xpress data

In order to confirm the TraDIS-*Xpress* data, several knockout mutants from the Keio mutant collection [20] were tested. The *tdk* mutants were chosen as these would be expected to show increased susceptibility to trimethoprim (Table1, Fig. 3d). In addition, the *dksA* and *truA* mutants were chosen as these would be expected to show reduced susceptibility to trimethoprim (Table 1, Fig. 3b). The TraDIS-*Xpress* data indicated that both *acrR* and *ompF* mutants would be likely to show no or very little change in trimethoprim susceptibility, and so these mutants also were chosen as controls. The Keio collection mutant pairs were compared with the parent strain from which the collection was derived (* E. coli* strain BW25113).

Initially, each chosen mutant was tested for trimethoprim MIC in quadruplicate. In all cases, the MIC difference between the mutant and BW25113 parent strain was less than two-fold. The MIC for the *tdk* mutants averaged at 0.2 mg l^−1^ compared to 0.3 mg l^−1^ for the parent strain, and 0.4 mg l^−1^ for the *truA* mutant whilst the MICs for *acrR* and *ompF* mutants averaged at 0.35 mg l^−1^. These were the largest differences observed between the parent strain and any of the mutants. Thus, whilst the MIC values for the *tdk* and *truA* mutants are trending higher or lower as expected, the differences are too small to provide a robust confirmation of the TraDIS data. This is not unexpected, as it has been demonstrated previously that the TraDIS method is very sensitive and capable of measuring phenotypic differences that are not measurable by MIC alone [19, 26, 27].

**Fig. 4.**
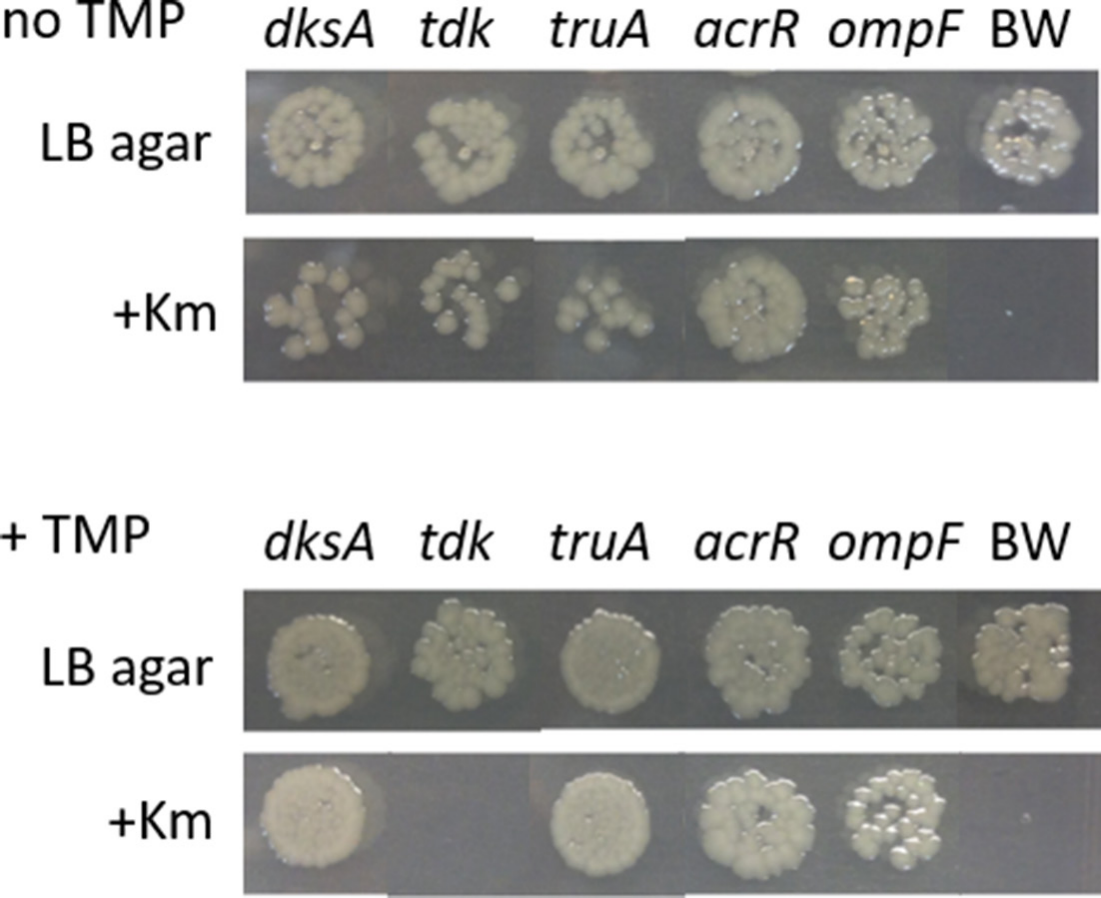
Results of growth competition between mutants and parent strain in the presence or absence of trimethoprim. Shown are colonies that grew following growth of mutants from the Keio collection [20] in competition with the parent strain BW25113 without trimethoprim (TMP, upper panel) and with 0.25 mg l^−1^ trimethoprim (lower panel). Approximately equal numbers of the parent strain and mutant to be tested were inoculated into LB broth and grown at 37 °C for 20 h. Then, each culture was serially diluted and 5 µl spotted onto LB-agar to allow growth of both parent strain and mutant, and onto LB-agar supplemented with kanamycin (Km) to allow growth of the mutant only. Following growth without trimethoprim, all mutants persisted in the cultures (upper panel). With 0.25 mg l^−1^ trimethoprim, the *tdk* mutant was lost from the culture, whilst the *dksA* and *truA* mutants dominated the culture (lower panel). As predicted from the TraDIS-*Xpress* data, trimethoprim made less, if any, obvious difference to growth of the *acrR* and *ompF* mutants. BW indicates a control culture with BW25113 alone. Mutants of the Keio collection come as independent pairs for each gene, and both mutants of the pairs were tested. The results were the same for each, so only one is presented.

As an alternative method to confirm the change in fitness the mutants were grown in competition with the parent strain in the presence and absence of a sub-MIC concentrations of trimethoprim to determine if they demonstrated a gain or loss of fitness compared with the parent strain. This competition assay resembles the conditions of the TraDIS-*Xpress* experiments where the mutants are all grown together, and relative fitness is determined. The *tdk* knockout mutants were lost from the cultures grown with trimethoprim but grew in comparable numbers to the competing BW25113 when trimethoprim was absent, whilst the *dksA* and *truA* knockout mutants grew to dominate over the parent strain in the presence of trimethoprim (Fig. 4). As expected, the *acrR* and *ompF* mutants showed no obvious difference between growth with and without trimethoprim (Fig. 4). Thus, these competition assays support the hypothesis that *tdk* mutants would display reduced fitness and the *dksA* and *truA* mutants increased fitness in the presence of trimethoprim, confirming the TraDIS-*Xpress* data. The MIC of trimethoprim that was determined for these mutants did not demonstrate any consistent significant differences between mutants and the parent strain. These findings highlight the precision of TraDIS-*Xpress* data (which is based on competitive fitness) and suggest that MIC-based approaches may be too insensitive to identify genotypic changes that result only in a small impact on antibiotic sensitivity, which may nonetheless have a large impact on fitness in competition.

### Increased transcription of target is a major mechanism for reduced susceptibility to trimethoprim, but not sulfamethoxazole

As well as providing gene insertional inactivation information, the outward transcribing promoter of the transposon used to generate the mutants can also assay genes by insertion upstream or downstream of genes. In this regard, the most statistically significant fitness advantage following exposure to trimethoprim was conferred by transposon insertion mutations 5′-to the *folA* gene (max log_2_FC of 5, *q*=6.5×10^−70^ at 2×MIC; Fig. 3a). All these insertion mutations were oriented such that the outward-transcribing promoter of the transposon were capable of transcribing the *folA* gene. This gene encodes DHFR which is inhibited by trimethoprim and the TraDIS-*Xpress* data indicate that increasing its expression reduces susceptibility to trimethoprim. This mechanism for reduced susceptibility to trimethoprim has been reported in clinical isolates of *E. coli* and has been created in the laboratory [3, 14], thus confirming that the TraDIS-*Xpress* technology is working as expected to identify possible drug-resistance mechanisms. These data demonstrate that TraDIS-*Xpress* technology has the ability to effectively assay essential genes (such as *folA*)*,* that cannot be identified by conventional transposon-mutagenesis approaches (Fig. 3a), as insertional mutations within essential genes are not obtained, so without the outward-transcribing transposon promoter, this gene would not have been identified.

The increase in insertion mutants 5′ to *folA* was obtained for growth in 2, 1, and 0.5× MIC of trimethoprim, but not at 0.25 × MIC where insertion mutations in several other loci provided a greater or similar growth advantage, indicating that fitness is dependent both on the mutation location and the antibiotic concentration.

Only three other genes were identified with 5′-insertions that potentially activate transcription resulting in reduced susceptibility to trimethoprim (using a filter of *q*<0.0001, log_2_FC>1.5 and log_2_CPM>5). These included *rraB* (max log_2_FC of 3 at 2×MIC, *q*=2.8×10^−5^), *intD* (max log_2_FC of 2.2 at 1×MIC, *q*=8.6×10^−6^) and *malQ* (max log_2_FC of 2 at 1×MIC, *q*=4.8×10^−5^).

Following growth with sulfamethoxazole compared to growth without, no genes were identified with significant 5´-transcription activating insertions that may result in reduced susceptibility . This included the *folP* gene coding for DHPS which is the enzyme target of sulfamethoxazole (using the same filter of *q*<0.0001, log_2_FC>1.5 and log_2_CPM>5). Thus, the data indicate that, unlike *folA* with trimethoprim, altered expression of *folP* did not confer increased fitness. This result was again as expected as it has also been shown that overexpression of *folP* does not reduce susceptibility to sulfamethoxazole [3].

### Insertion mutations within genes that contribute to reduced trimethoprim and/or sulfamethoxazole susceptibility

During growth in the presence of trimethoprim or sulfamethoxazole, relative numbers of transposon insertion mutants for some genes increased, indicating that these mutants had a selective advantage in trimethoprim and or sulfamethoxazole. These genes, therefore, represent candidates for which loss of function could contribute to resistance in clinical isolates. Thirty genes presented in Table 1 are of this category and of these *rpoN*, *fis*, *glnA*, *fbp*, *dnaJ*, *dsbA* and *sthA* for trimethoprim and *prmB*, *glyA* and *ychF* for sulfamethoxazole gave the greatest fold changes in the proportion of mutants (Table 1, Fig. 1). Transposon insertion mutations in eight genes resulted in an increased growth advantage both in the presence of trimethoprim and of sulfamethoxazole, and of these the greatest fold changes in proportion of mutants was observed for *dcd*, *prfC*, *hfq*, *ygdI*, *truA* and *rapA* (Table 1).

### Insertion mutations within genes that contribute to increased trimethoprim and/or sulfamethoxazole susceptibility

Following growth in the presence of trimethoprim or sulfamethoxazole, insertion mutants for a set of genes showed a relative decrease in numbers, indicating their increased susceptibility to these antibiotics. Of these genes, the ones for which mutant loss was the greatest following growth in trimethoprim were *tdk* (encoding thymidine kinase, Fig. 3d), *deoR*, *ybbC*, *ldcA*, *hha*, and *wbbK*, whilst for sulfamethoxazole, *rsmH*, *fadR*, *ddlB*, *nlpI* and *wzxE* (Fig. 3e) mutants showed the greatest relative decrease (Table 1). Mutants with transposon insertions in *ispD*, *uspC*, *minD*, *minC*, *yebK*, *truD*, and *umpG* showed significantly reduced fitness following growth both with trimethoprim and with sulfamethoxazole (Table 1).

### Inhibition of tdk synergises with trimethoprim

TraDIS-*Xpress* data predicts antibacterial synergy of trimethoprim together with a Tdk inhibitor. It has been shown previously that 3′-azido-3′-deoxythymidine (AZT) inhibits Tdk [28]. The *tdk* gene is not essential and so mutants that lack the Tdk target are resistant to AZT. The MIC of AZT against *tdk*-knockout mutants from the Keio collection confirmed that the mutants had an MIC of over 10 mg l^−1^ compared to the parent strain BW25113 with an MIC of 0.00625 mg l^−1^. The MIC of trimethoprim was determined in the presence of a range of AZT concentrations and confirmed the predicted synergy, as has been found previously [29]. Thus, in the presence of 0.0016 mg l^−1^ AZT, the MIC of TMP was 0.0625, a 16-fold reduction. The fractional inhibitory concentration index for the pair was determined as 0.375, confirming synergy [29].

AZT is a nucleoside with antiviral activity and has been used to treat HIV patients. This raises the possibility that other nucleoside-based drugs may also possess antibacterial activity. Other nucleoside-based antiviral agents were therefore tested for antibacterial activity, including Stavudine, Ganciclovir and 2’,3’-dideoxyinosine (ddI). However, none of these nucleosides showed any significant antibacterial activity, with MICs measured at 280 mg l^−1^ for Stavudine, 624 mg l^−1^ for ddI and over 1200 mg l^−1^ for Ganciclovir.

## Discussion

The work presented here describes the application of TraDIS-*Xpress* to investigate susceptibility mechanisms to trimethoprim and sulfamethoxazole. The target of trimethoprim action is the dihydrofolate reductase, FolA, increased expression of which is a known resistance mechanism [14], and our data identified this. However, this was not the case for sulfamethoxazole whose target is the *folP* gene product and for which over-expression is known not to be a resistance mechanism [3]. Thus, whilst TraDIS-*Xpress* may provide clues to the identity of the unknown targets for some antibacterial compounds, it also has limitations in this application [30]. However, we were able to identify and validate with high sensitivity several genes involved in susceptibility to both drugs, demonstrating the power of this approach to reveal a range of genes and pathways involved in responses to antimicrobials.

Previously, a chemical-genomic data set was generated by subjecting 3979 mutants from the Keio collection to 324 different growth conditions, including 139 antibiotics [31]. Data sets such as these have proven useful, including the finding that trimethoprim and the antiviral nucleoside AZT, which inhibits the thymidine kinase Tdk, act synergistically as antibiotics against *E. coli* [29]. Our TraDIS-*Xpress* experiments identified that *tdk* mutants have reduced fitness in the presence of trimethoprim, leading us to confirm this previously observed trimethoprim-AZT synergy. In addition, our data identified that knockout mutations in other genes, including *deoR*, *ybbC*, *ldcA*, *hha*, and *wbbK,* also conferred reduced fitness with trimethoprim (Table 1), and likewise for sulfamethoxazole, including the *rsmH*, *fadR*, *ddlB*, *nlpI* and *prc* genes. Knockout of *ispD*, *uspC*, *minD*, *minC*, *yebK*, *truD*, or *umpG* genes conferred reduced fitness in both these antibiotics. Chemical compounds that inhibit these gene products may provide synergistic antibacterial activity with trimethoprim, sulfamethoxazole and/or cotrimoxazole. Thus, TraDIS-*Xpress* can provide a rational approach to identifying all of the likely target candidates of synergy with a chosen antibiotic.

Previous work has shown that *tdk*, *deoR* and *ldcA* mutants grow more poorly than the parent strain in the presence of trimethoprim, confirming our results for these genes [31]. It has also been shown previously that fosmidomycin, an inhibitor of IspD, shows synergistic antibacterial activity with trimethoprim [32, 33], confirming our finding that growth of *ispD* mutants is compromised in the presence of trimethoprim. Thus, in a single set of parallel experiments, we were able to identify two known examples of synergy between trimethoprim and other antibacterial compounds. This confirms that products of the other genes that we, and in some cases previous work, have identified represent an extended set of candidate targets for chemical compounds that synergise with trimethoprim, sulfamethoxazole and/or cotrimoxazole.

The advantages of TraDIS-*Xpress* over previous methods used to generate chemical-genetic datasets are that all mutants are generated simultaneously and are then all tested together for each condition. This removes the labour-intensive step of making and systematically storing many individual defined mutants. Furthermore, the number of experiments is limited to the number of different conditions under investigation, rather than the product of the number of mutants to be tested and the number of conditions. Thus, the generation of several chemical-genomics data sets can be more rapid. In addition, previous chemical-genomic data sets have used a limited number of knockout mutants per gene (from the ‘Keio collection’; [20, 31]). Our collection includes knockout and altered expression mutants and, with a transposon insertion site on average every 10 bp across the entire bacterial genome, provides a high resolution, information-rich dataset unbiased by known locations of genes. In addition, previous datasets have measured growth from colony size [31], whilst TraDIS-*Xpress* measures growth from the number of sequence reads, which reflects the number of mutants and is likely to detect more subtle growth differences. We were able to identify and, in some cases, validate minor roles for genes which would not be detectable without this level of sensitivity. Whilst such subtle mechanisms that reduce antibiotic susceptibility are unlikely to contribute to antibacterial resistance, they may when in combination with each other, and they may complement other resistance determinants. Whole genome analyses that seek to identify mechanisms of resistance to these antibiotics in clinical isolates should therefore include close attention to these genes. These subtle mechanisms also provide insights into the general effects that antibiotics have on bacterial cells.

Using TraDIS-*Xpress* we have independently identified and confirmed that an inhibitor of thymidine kinase, AZT, and trimethoprim act synergistically as antibacterials, and predicted the known synergy between trimethoprim and the inhibitor of the IspD (4-diphosphocytidyl-2C-methyl-d-erythritol synthase) enzyme, fosmidomycin [32]. Also, we have identified additional candidate targets, inhibitors of which may act synergistically with trimethoprim, sulfamethoxazole or both. In addition, most of the genes identified by our analysis as contributing to trimethoprim and sulfamethoxazole susceptibility encode metabolic functions that are unrelated to DHFR and DHPS, and to each other, demonstrating how the technology can identify relevant genes that would otherwise not have been predicted. Thus, the data demonstrate the value of TraDIS-*Xpress* for antibacterial research and development.

## Supplementary Data

Supplementary material 2
